# IgG antibodies label the spermatozoan centriole of Drosophila melanogaster

**DOI:** 10.17912/micropub.biology.000838

**Published:** 2023-05-26

**Authors:** Ankit Jaiswal, Tomer Avidor-Reiss

**Affiliations:** 1 Biological Sciences, University of Toledo, Toledo, Ohio, United States

## Abstract

Centrioles are microtubule-based, barrel-shaped, subcellular organelles with evolutionarily conserved composition, structure, and function. Yet, in sperm cells, centrioles are remodeled, gaining species-specific composition and structure. The sperm centrioles of *Drosophila melanogaster* undergo dramatic remodeling, during which most known centriolar proteins are lost. Here, we find that *IgG antibodies unexpectedly label Drosophila melanogaster spermatozoan centrioles* . This labeling offers a simple method for marking the spermatozoan centriole, though it may interfere with testing new anti-centriolar antibodies using immunofluorescence.

**Figure 1. IgG antibodies made in donkey and goat, but not chicken IgY nor alpaca VHH fragment, label the spermatozoon centriole of Drosophila melanogaster:**
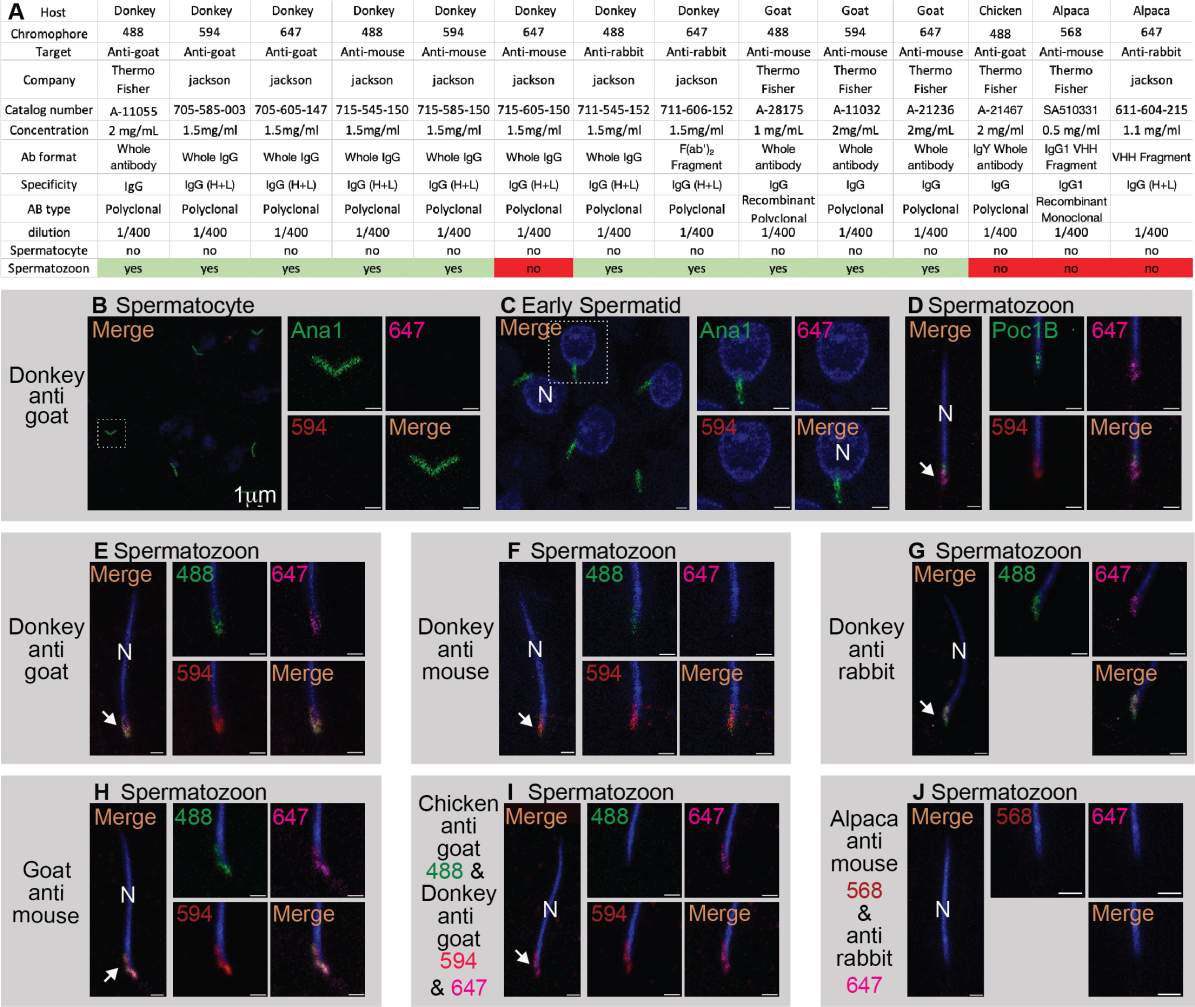
A) A table summarizing the properties, dilution, and localization in spermatocyte and spermatozoon of the tested antibodies. B–J) Labeling of Spermatocyte (B), Early Spermatid (C), and Spermatozoon (D–J) by the antibodies described in “A”. Transgenic flies expressing Ana1-GFP driven by the Ana1 promoter were used in “B” and “C”, while flies expressing Poc1B-GFP driven by the Bam-Gal4 promoter were used in “D”. Control *w ^1118^* flies were in “E” through “J”. Each panel includes a low-magnification figure on the left. A dotted square marks the magnified centriole in the panels to the right in “B” and “C”. An arrow marks the centriolar region found at the base of the nucleus (N, blue). Each staining was repeated at least three times with similar results. Scale bars are 1 mm in all panels.

## Description

Spermatozoa are highly specialized cells with unique compositions, structures, and functions. One of these specialized spermatozoan structures is the centriole. Unlike in most cells, which contain centrioles with canonical structure and composition, in spermatozoa, one or both centrioles have atypical structure and composition (reviewed in Avidor-Reiss and Fishman 2019). These atypical centrioles are formed when spermatid canonical centrioles are remodeled (aka centrosome reduction) during spermiogenesis (reviewed in Manandhar et al. 2005).

Centrioles are ancient, eukaryotic, subcellular structures with a high level of evolutionary conservation and a set of evolutionarily conserved proteins, including Sas-6, CPAP/SAS-4, Cep52/ASL, CEP135/BLD10, and Cep295/ANA1 (Carvalho-Santos et al. 2011) . One of the most dramatic remodeling of sperm centrioles occurs in Fruit fly (Drosophila melanogaster), in which all these proteins are stripped away (Riparbelli et al. 2010) . For this reason, Drosophila is commonly used as a model system for studying centriole biology and the sperm centrosome (Khire et al. 2015) . Most Drosophila centriolar proteins–such as CEP135/BLD10, HYLS1, and Sas-6–are observed in early spermatids but are undetected in late spermatids or in spermatozoa stored in the seminiferous tubules (Rodrigues-Martins et al. 2007; Mottier-Pavie and Megraw 2009; Hou et al. 2020) . Currently, the only centriolar proteins known to be present in fly spermatozoa are the spliced isoforms of Poc1, Poc1A and Poc1B, (Khire et al. 2016) . This lack of detection in spermatozoa of other centriolar proteins is due to centriole remodeling, which strips them from the centrioles during spermiogenesis. The absence of so many centriolar proteins raises the question as to which sperm centriolar proteins form the zygotic centrosome post-fertilization.

During our studies to identify new components of fly spermatozoan centrioles using indirect immunofluorescence, we found that many IgG proteins used as secondary antibodies directly label the sperm centriole or its vicinity (Fig 1). We found that this labeling provides both advantages and disadvantages: while commercial, fluorophore-labeled secondary antibodies offer a simple method for marking the spermatozoan centriole, one disadvantage is that they interfere with testing the labeling of spermatozoan centrioles by anti-centriolar antibodies. Therefore, here, we have characterized the labeling of fly spermatozoa using antibodies conjugated with various chromophores from several shared hosts and with several targets and specificities (Fig 1A).

Donkey secondary antibodies are a standard tool, and, therefore, we first aimed to identify the labeling pattern of Donkey whole IgG made against Goat IgG heavy and light chains (H+L). To determine centriolar location, we used transgenic flies that express the centriole-specific protein Ana1-GFP (Blachon et al. 2009) or Poc1B-GFP (Khire et al. 2016) . We found that Donkey IgG made against Goat IgG and conjugated with Alexa 594 or 647 does not label the centrioles of spermatocytes (Fig 1B) nor those of early spermatids (Fig 1C). However, it labeled the base of the nucleus, partly overlapping with the centrioles of spermatozoa found in the seminal vesicle (Fig 1D–E).

Next, we aimed to identify the target species specificity of Donkey secondary antibodies. We found that the spermatozoan centriolar region is labeled by commercial Donkey whole IgG made against mouse IgG and conjugated with either Alexa 488 or Alexa 594 but is not labeled by commercial Donkey whole IgG conjugated with Alexa 647 (Fig 1F). The spermatozoan centriolar region is also labeled by Donkey whole IgG made against rabbit IgG and conjugated with either Alexa 488 or Alexa 647 (Fig 1G). These findings demonstrate that Donkey antibodies against IgG of various target mammals label the fly spermatozoan centrioles.

Next, we aimed to identify the host species specificity of this labeling. We found that commercial Goat IgG made against mouse IgG and conjugated with either Alexa 488, Alexa 594, or Alexa 647 labels the spermatozoan centriolar region (Fig 1H). The spermatozoan centriolar region is not labeled by commercial Chicken IgY made against goat IgG and conjugated with Alexa 488 (Fig 1I). Finally, we tested Alpaca nanobodies conjugated with either Alexa 568 or 647. These are distinct antibodies made of heavy-chain variable domains (VHH fragment) (Reviewed in Asaadi et al. 2021). The nanobodies did not label the centriolar region (Fig 1J). These findings suggest that IgG antibodies of mammals but not chicken IgY nor Alpaca VHH fragment can recognize the fly spermatozoan centrioles.

Most IgG secondary antibodies cross-react with centrioles, except alpaca VHH fragment and chicken IgY. It appears that the antibody's binding ability to the fly sperm centriole is either due to the alpaca or chicken origin, or, alternatively, due to the fragmentary nature of the alpaca antibody. Therefore, we concluded that it would be best to use Chicken IgY or Alpaca VHH fragment secondary antibodies in immunofluorescent microscopy studies of spermatozoan centrioles to reduce the chances of direct labeling of the spermatozoan centrioles by secondary antibodies. A potential explanation for the affinity of many, but not all, mammalian IgG antibodies to the fly spermatozoan centrioles is that the IgG of many mammals may have a shared (with some variations) amino acid sequence or 3-D organization that binds a fly spermatozoan protein found at or near the centriole.

## Methods

For antibody staining, testes of adult fruit flies were dissected in PBS, squashed with a coverslip, frozen in liquid nitrogen for 5 minutes, and fixed in ice-cold methanol (stored at -20 °C) for 5 minutes at room temperature. Slides were then washed with PBS in a slotted glass Coplin jar for one minute, followed by permeabilization with 0.1% Triton X-100 in PBS in a slotted glass Coplin jar for 30 minutes and blocking with 1% bovine serum albumin (BSA) in PBS with 0.1% Triton X-100 in a slotted glass Coplin jar for 30 minutes. 100 μL of secondary antibodies diluted in PBS with 1% BSA and 0.1% Triton X-100 were applied to each slide, which was then covered with Parafilm and incubated overnight at 4 °C.

The next day, slides were washed three times in PBS with 0.1% Triton X-100 in a slotted glass Coplin jar for five minutes per wash. Samples were then washed three times for five minutes per wash in PBS in a slotted glass Coplin jar, and excess PBS was dabbed off of each slide using Kimwipes following the final wash. Then, one drop of Fluoroshield with DAPI was added over the testis sample on the slide, glass coverslips were placed over each sample, and each coverslip was sealed with clear nail polish. Slides were kept at -20 °C until analysis.

Slides were visualized using a Leica SP8 confocal microscope in BrightR mode using an HC PL APO CS2 63x/1.40 OIL lens, 100% gain, 4096 × 4096 pixel (245 μm × 245 μm) format, 0.75 zoom factor, 2.0 frame accumulation, and rotation set at 90.00. The fluorescence signal was collected using four sequences. Sequence one capture DNA staining via DAPI, for this we activated a 410-nm (UV) laser at 0.1% power. The absorption spectrum was set to cover 412–474 nm via the HyD1 detector and was assigned blue. To capture the GFP signal or ALEXA 488 signal (sequence two), we activated a 488-nm laser set at 4% power. The absorption spectrum was set to cover 506–545 nm via the HyD3 detector and was assigned green. To capture ALEXA 594 or 568 (sequence three), we activated a 561-nm laser set at 4% power. The absorption spectrum was set to cover 600–641 nm for Alexa 594 and 580–628 nm for Alexa 568 via the HyD3 detector and was assigned red. To capture ALEXA 647 (sequence four), we activated a 633-nm laser set at 4% power. The absorption spectrum was set to cover 651–695 nm via the HyD4 detector and was assigned magenta. We collected multiple (10−20) Z-sections of 0.3 μm thickness from the top of the highest sperm to the bottom of the lowest sperm and showed the projection in the figure.

## Reagents

Solutions:

Washing Solution: PBS - 1000 ml made in double-distilled water (8 gm NaCl, 0.2 gm KCl, 1.44 gm Na _2_ HPO _4_ , 0.24 gm KH _2_ PO _4_ , pH 7.4).

Permeabilization Solution: PBS with 0.1% Triton X-100 (PBST) (Sigma Aldrich, 9002-93-1)

Blocking Solution: PBST with 1% BSA (PBSTB) (CHEM-IMPEX INT’L, 00535)

Fixation: Methanol (stored at -20 °C) (Fisher Chemical, A412P-4)

Materials:

Confocal Microscope: Leica SP8; images were captured at a magnification of 640x and 6x zoom at 512 × 512 pixel density, 3x zoom at 1024 × 1024 pixel density, or 0.75x zoom at 4096 × 4096 pixel density.

Using Photoshop, immunofluorescence sperm images were rotated, reoriented, and cropped to:

Medium Zoom, 800 × 800 pixels, adjusted to 2 × 2 inches (Spermatocyte); Medium Zoom, 300 × 300 pixels, adjusted to 2 × 2 inches (Early Spermatid); Medium Zoom, 100 × 250 pixels, adjusted to 2 × 5 inches (Spermatozoa); zoomed-in pictures of the sperm neck were cropped to 100 × 100 pixels, then adjusted to 2 × 2 inches.

Adobe Illustrator was used to create the final figure with annotations.

Triton X-100 (Sigma-Aldrich, 9002–93–1)

BSA (CHEM-IMPEX INT’L, 00535)

Parafilm (Bemis™, 13–374–12)

Kimwipes (34155/EMD)

Cover Slip (Epredia, 152440)

Slides (Azer Scientific, EMS200A+)

Nail Polish (EMS Diasum, 72180)

Mounting Media: Fluoroshield with DAPI (Sigma-Aldrich, F6057-20ML)

LAS X Software Leica using BrightR.

Experiments were performed 3 to 4 times.

**Fly Strains:**

**Table:** 

**Strain**	**Genotype**	**Available from**
Ana1-GFP	w; Ana1-GFP; MKRS/TM6B	Avidor-Reiss lab
Poc1B-GFP	BAM; Cyo/sp; UAS-Poc1B-GFP/TM6B	Avidor-Reiss lab
w ^1118^ (BDSC stock 3605)	w ^1118^	Bloomington Drosophila Stock Center

**Table:** 
